# 1-(2-Azido­acet­yl)-3-methyl-2,6-diphenyl­piperidin-4-one

**DOI:** 10.1107/S1600536810039097

**Published:** 2010-10-09

**Authors:** Haldorai Yuvaraj, S. Sundaramoorthy, D. Velmurugan, Rajesh G. Kalkhambkar

**Affiliations:** a School of Display and Chemical Engineering, Yeungnam University, Gyeongsan, Gyeongbuk 712-749, Republic of Korea; b Centre of Advanced Study in Crystallography and Biophysics, University of Madras, Guindy Campus, Chennai 600 025, India; c Department of Chemistry, Karnatak University, Karnatak Science College, Dharwad 580 001, Karnataka, India

## Abstract

In the title compound, C_20_H_20_N_4_O_2_, the piperidine ring adopts a distorted boat conformation. The two phenyl rings form dihedral angles of 82.87 (1) and 84.40 (1)° with respect to the piperidine ring. The crystal packing is stabilized by inter­molecular C—H⋯O and C—H⋯N inter­actions.

## Related literature

For the biological activity of piperidines, see: Aridoss *et al.* (2008 ▶, 2010 ▶). For ring conformational analysis, see: Cremer & Pople (1975 ▶); Nardelli (1983 ▶). For related structures, see: Jeyaraman *et al.* (1999 ▶); Keana & Cai (1990 ▶); Ponnuswamy *et al.* (2002 ▶).
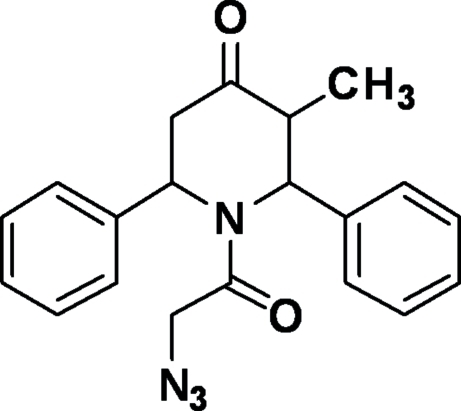


## Experimental

### 

#### Crystal data

C_20_H_20_N_4_O_2_
*M* *_r_* = 348.40
Monoclinic, 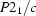

*a* = 11.0418 (3) Å
*b* = 15.7844 (5) Å
*c* = 10.5684 (3) Å
β = 108.458 (2)°
*V* = 1747.19 (9) Å^3^
*Z* = 4
Mo *K*α radiation
μ = 0.09 mm^−1^
*T* = 292 K
0.25 × 0.23 × 0.2 mm

#### Data collection

Bruker SMART APEXII area-detector diffractometer
Absorption correction: multi-scan (*SADABS*; Bruker, 2008 ▶) *T*_min_ = 0.978, *T*_max_ = 0.983
16435 measured reflections
4286 independent reflections
3147 reflections with *I* > 2σ(*I*)
*R*_int_ = 0.026

#### Refinement

*R*[*F* ^2^ > 2σ(*F* ^2^)] = 0.045
*wR*(*F* ^2^) = 0.138
*S* = 1.05
4286 reflections
236 parameters
H-atom parameters constrained
Δρ_max_ = 0.24 e Å^−3^
Δρ_min_ = −0.18 e Å^−3^

### 

Data collection: *APEX2* (Bruker, 2008 ▶); cell refinement: *SAINT* (Bruker, 2008 ▶); data reduction: *SAINT*; program(s) used to solve structure: *SHELXS97* (Sheldrick, 2008 ▶); program(s) used to refine structure: *SHELXL97* (Sheldrick, 2008 ▶); molecular graphics: *ORTEP-3* (Farrugia, 1997 ▶); software used to prepare material for publication: *SHELXL97* and *PLATON* (Spek, 2009 ▶).

## Supplementary Material

Crystal structure: contains datablocks global, I. DOI: 10.1107/S1600536810039097/bt5365sup1.cif

Structure factors: contains datablocks I. DOI: 10.1107/S1600536810039097/bt5365Isup2.hkl

Additional supplementary materials:  crystallographic information; 3D view; checkCIF report

## Figures and Tables

**Table 1:** Hydrogen-bond geometry (Å, °)

*D*—H⋯*A*	*D*—H	H⋯*A*	*D*⋯*A*	*D*—H⋯*A*
C9—H9⋯O1^i^	0.93	2.57	3.464 (2)	162
C5—H5⋯N2^ii^	0.98	2.52	3.353 (2)	142
C2—H2*B*⋯O2^ii^	0.97	2.56	3.4933 (19)	161

Symmetry codes: (i) 
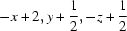
; (ii) 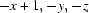.

**Fig. 1.**
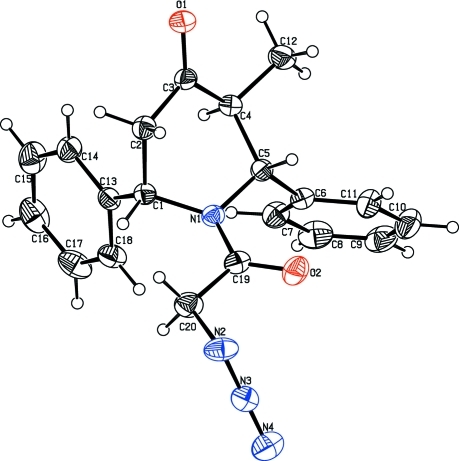
Perspective view of the molecule showing the thermal ellipsoids drawn at 30% probability level.

**Fig. 2.**
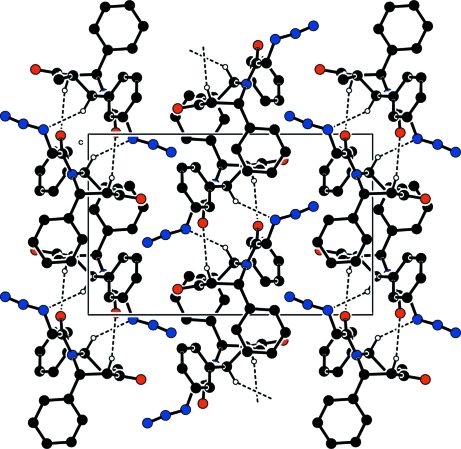
The crystal packing of the molecules viewed down a axis. For clarity, hydrogen atoms which are not involved in hydrogen bonding are omitted

## supplementary crystallographic information

### Comment

2,6-Diarylpiperidin-4-ones normally adopt chair conformation with equatorial orientation of all the substituents. Nevertheless, introduction of some heteroconjugate groups such as –NO, –CHO, –COCH_3_, –COC_6_H_5_, *etc*., at the heteroatom of 2,6-disubstitutedpiperidine ring system have reported to cause a major change in ring conformation, and orientation of the substituents (Jeyaraman *et al.*, 1999; Ponnuswamy *et al.*, 2002). To establish the conformational impact of azidoacetyl group at the nitrogen of 2,6-diphenyl -3-methylpiperidin-4-one, the current study has been undertaken.

The *ORTEP* plot of the title molecule is shown in Fig.1. The piperidine ring adopts a distorted boat conformation with the puckering parameters (Cremer & Pople, 1975) and asymmetry parameters (Nardelli, 1983) of q_2_ = 0.699 (5) Å, q_3_ = -0.043 (2) Å, φ_2_ = 68.242 (8)° and Δ_s_ (C1 and C4) = 9.5 (2) Å. The sum of the bond angles around the atom N1[(355.42 (3)°] of the piperidine ring is in accordance with the *sp*^2^ hybridization.

The crystal structure is stabilized by C—H···O and C—H···N intermolecular interactions which link the molecules into chains running along the *c* axis. Atoms C2 and C5 at (*x*, *y*, *z*) donate one proton to acceptor O2 and N2 at (-*x* + 1, -*y*, -*z*), forming a centrosymmetric dimers (Fig. 2) with *R_2_^2^*(12) ring motifs.

### Experimental

1-(2-Azidoacetyl)-3-methyl-2,6-diphenylpiperidin-4-one was prepared from the reaction of 1-chloroacetyl-3-methyl-2,6-diphenylpiperidin -4-one with NaN_3_ as per the reported procedure (Keana & Cai, 1990). The obtained crude mass was purified by column chromatography followed by recrystallization from ethanol giving colourless, diffraction quality crystals.

### Refinement

The C bound H atoms positioned geometrically (C—H = 0.93–0.98 Å) and allowed to ride on their parent atoms, with 1.5*U*_eq_(C) for methyl H and 1.2 *U*_eq_(C) for other H atoms.

### Crystal data

**Table:**

C_20_H_20_N_4_O_2_	*F*(000) = 736
*M**_r_* = 348.40	*D*_x_ = 1.324 Mg m^−^^3^
Monoclinic, *P*2_1_/*c*	Mo *K*α radiation, λ = 0.71073 Å
Hall symbol: -P 2ybc	Cell parameters from 1520 reflections
*a* = 11.0418 (3) Å	θ = 1.9–28.5°
*b* = 15.7844 (5) Å	µ = 0.09 mm^−^^1^
*c* = 10.5684 (3) Å	*T* = 292 K
β = 108.458 (2)°	Block, colourless
*V* = 1747.19 (9) Å^3^	0.25 × 0.23 × 0.2 mm
*Z* = 4	

### Data collection

**Table:**

Bruker SMART APEXII area-detector diffractometer	4286 independent reflections
Radiation source: fine-focus sealed tube	3147 reflections with *I* > 2σ(*I*)
graphite	*R*_int_ = 0.026
ω and φ scans	θ_max_ = 28.5°, θ_min_ = 1.9°
Absorption correction: multi-scan (*SADABS*; Bruker, 2008)	*h* = −14→14
*T*_min_ = 0.978, *T*_max_ = 0.983	*k* = −19→21
16435 measured reflections	*l* = −14→13

### Refinement

**Table:**

Refinement on *F*^2^	Primary atom site location: structure-invariant direct methods
Least-squares matrix: full	Secondary atom site location: difference Fourier map
*R*[*F*^2^ > 2σ(*F*^2^)] = 0.045	Hydrogen site location: inferred from neighbouring sites
*wR*(*F*^2^) = 0.138	H-atom parameters constrained
*S* = 1.05	*w* = 1/[σ^2^(*F*_o_^2^) + (0.0678*P*)^2^ + 0.3241*P*] where *P* = (*F*_o_^2^ + 2*F*_c_^2^)/3
4286 reflections	(Δ/σ)_max_ < 0.001
236 parameters	Δρ_max_ = 0.24 e Å^−^^3^
0 restraints	Δρ_min_ = −0.18 e Å^−^^3^

### Special details

**Table:**

Geometry. All e.s.d.'s (except the e.s.d. in the dihedral angle between two l.s. planes) are estimated using the full covariance matrix. The cell e.s.d.'s are taken into account individually in the estimation of e.s.d.'s in distances, angles and torsion angles; correlations between e.s.d.'s in cell parameters are only used when they are defined by crystal symmetry. An approximate (isotropic) treatment of cell e.s.d.'s is used for estimating e.s.d.'s involving l.s. planes.
Refinement. Refinement of *F*^2^ against ALL reflections. The weighted *R*-factor *wR* and goodness of fit *S* are based on *F*^2^, conventional *R*-factors *R* are based on *F*, with *F* set to zero for negative *F*^2^. The threshold expression of *F*^2^ > σ(*F*^2^) is used only for calculating *R*-factors(gt) *etc*. and is not relevant to the choice of reflections for refinement. *R*-factors based on *F*^2^ are statistically about twice as large as those based on *F*, and *R*- factors based on ALL data will be even larger.

### Fractional atomic coordinates and isotropic or equivalent isotropic displacement parameters (Å^2^)

**Table:**

	*x*	*y*	*z*	*U*_iso_*/*U*_eq_	
C1	0.59213 (13)	0.02968 (9)	0.33092 (13)	0.0366 (3)	
H1	0.5050	0.0521	0.3073	0.044*	
C2	0.57944 (14)	−0.06647 (9)	0.32572 (15)	0.0411 (3)	
H2A	0.5489	−0.0853	0.3976	0.049*	
H2B	0.5166	−0.0827	0.2421	0.049*	
C3	0.70355 (14)	−0.10988 (9)	0.33785 (14)	0.0392 (3)	
C4	0.80956 (13)	−0.05375 (9)	0.32448 (14)	0.0386 (3)	
H4	0.8441	−0.0228	0.4087	0.046*	
C5	0.75819 (13)	0.01278 (9)	0.21275 (13)	0.0360 (3)	
H5	0.7324	−0.0182	0.1281	0.043*	
C6	0.86401 (14)	0.07294 (9)	0.20829 (14)	0.0387 (3)	
C7	0.92891 (16)	0.12263 (10)	0.31632 (17)	0.0498 (4)	
H7	0.9025	0.1239	0.3916	0.060*	
C8	1.03309 (18)	0.17048 (12)	0.3130 (2)	0.0638 (5)	
H8	1.0764	0.2034	0.3863	0.077*	
C9	1.07271 (18)	0.16958 (12)	0.2027 (2)	0.0661 (5)	
H9	1.1430	0.2015	0.2012	0.079*	
C10	1.00821 (19)	0.12146 (13)	0.0946 (2)	0.0670 (5)	
H10	1.0341	0.1211	0.0190	0.080*	
C11	0.90476 (17)	0.07345 (11)	0.09768 (17)	0.0536 (4)	
H11	0.8617	0.0408	0.0238	0.064*	
C12	0.91822 (16)	−0.10347 (11)	0.30249 (19)	0.0556 (4)	
H12A	0.9447	−0.1471	0.3688	0.083*	
H12B	0.9887	−0.0661	0.3093	0.083*	
H12C	0.8903	−0.1287	0.2154	0.083*	
C13	0.66501 (13)	0.06839 (9)	0.46567 (14)	0.0386 (3)	
C14	0.70059 (15)	0.02104 (11)	0.58243 (15)	0.0489 (4)	
H14	0.6877	−0.0373	0.5783	0.059*	
C15	0.75481 (18)	0.05956 (14)	0.70438 (17)	0.0639 (5)	
H15	0.7784	0.0269	0.7816	0.077*	
C16	0.77431 (18)	0.14548 (15)	0.71305 (19)	0.0683 (6)	
H16	0.8088	0.1713	0.7958	0.082*	
C17	0.74226 (18)	0.19344 (12)	0.5977 (2)	0.0645 (5)	
H17	0.7572	0.2515	0.6025	0.077*	
C18	0.68785 (16)	0.15484 (10)	0.47485 (17)	0.0506 (4)	
H18	0.6664	0.1874	0.3976	0.061*	
C19	0.56623 (15)	0.09208 (9)	0.10767 (14)	0.0416 (3)	
C20	0.43631 (16)	0.12667 (11)	0.10659 (16)	0.0531 (4)	
H20A	0.3892	0.0822	0.1339	0.064*	
H20B	0.4493	0.1726	0.1705	0.064*	
N1	0.64214 (11)	0.05607 (7)	0.22287 (11)	0.0364 (3)	
N2	0.36126 (14)	0.15755 (9)	−0.02523 (14)	0.0570 (4)	
N3	0.38863 (12)	0.22845 (9)	−0.05698 (13)	0.0475 (3)	
N4	0.39979 (17)	0.29178 (11)	−0.09966 (16)	0.0670 (4)	
O1	0.71688 (12)	−0.18542 (7)	0.35828 (12)	0.0547 (3)	
O2	0.59960 (12)	0.09701 (8)	0.00863 (11)	0.0566 (3)	

### Atomic displacement parameters (Å^2^)

**Table:**

	*U*^11^	*U*^22^	*U*^33^	*U*^12^	*U*^13^	*U*^23^
C1	0.0380 (7)	0.0361 (7)	0.0357 (7)	0.0033 (5)	0.0116 (6)	0.0017 (6)
C2	0.0417 (7)	0.0383 (8)	0.0434 (8)	−0.0047 (6)	0.0138 (6)	−0.0010 (6)
C3	0.0512 (8)	0.0315 (7)	0.0369 (7)	0.0001 (6)	0.0168 (6)	0.0005 (6)
C4	0.0421 (7)	0.0328 (7)	0.0418 (8)	0.0028 (5)	0.0146 (6)	0.0015 (6)
C5	0.0416 (7)	0.0327 (7)	0.0337 (7)	−0.0015 (5)	0.0119 (6)	−0.0024 (5)
C6	0.0421 (7)	0.0339 (7)	0.0391 (7)	−0.0004 (5)	0.0114 (6)	0.0043 (6)
C7	0.0534 (9)	0.0450 (9)	0.0493 (9)	−0.0079 (7)	0.0137 (7)	−0.0027 (7)
C8	0.0559 (10)	0.0492 (10)	0.0768 (13)	−0.0137 (8)	0.0077 (9)	−0.0012 (9)
C9	0.0519 (10)	0.0532 (10)	0.0941 (15)	−0.0071 (8)	0.0244 (10)	0.0206 (10)
C10	0.0680 (12)	0.0710 (13)	0.0728 (13)	−0.0020 (10)	0.0375 (11)	0.0185 (10)
C11	0.0605 (10)	0.0582 (10)	0.0459 (9)	−0.0059 (8)	0.0222 (8)	0.0021 (7)
C12	0.0505 (9)	0.0487 (9)	0.0719 (11)	0.0104 (7)	0.0254 (9)	0.0090 (8)
C13	0.0379 (7)	0.0405 (8)	0.0376 (7)	0.0066 (5)	0.0124 (6)	−0.0014 (6)
C14	0.0516 (9)	0.0536 (10)	0.0410 (8)	0.0025 (7)	0.0139 (7)	0.0029 (7)
C15	0.0606 (11)	0.0864 (15)	0.0393 (9)	0.0048 (9)	0.0083 (8)	0.0006 (9)
C16	0.0568 (10)	0.0925 (16)	0.0474 (10)	0.0053 (10)	0.0049 (8)	−0.0255 (10)
C17	0.0606 (11)	0.0529 (10)	0.0714 (13)	0.0038 (8)	0.0087 (9)	−0.0231 (9)
C18	0.0559 (9)	0.0409 (8)	0.0501 (9)	0.0066 (7)	0.0099 (7)	−0.0051 (7)
C19	0.0483 (8)	0.0361 (7)	0.0344 (7)	−0.0034 (6)	0.0044 (6)	−0.0004 (6)
C20	0.0542 (9)	0.0537 (10)	0.0445 (9)	0.0093 (7)	0.0057 (7)	0.0037 (7)
N1	0.0403 (6)	0.0343 (6)	0.0330 (6)	0.0011 (5)	0.0093 (5)	0.0010 (5)
N2	0.0614 (8)	0.0418 (8)	0.0505 (8)	0.0023 (6)	−0.0070 (7)	0.0026 (6)
N3	0.0451 (7)	0.0496 (8)	0.0402 (7)	0.0029 (6)	0.0029 (6)	−0.0050 (6)
N4	0.0798 (11)	0.0576 (10)	0.0584 (10)	−0.0084 (8)	0.0145 (8)	0.0053 (8)
O1	0.0714 (8)	0.0328 (6)	0.0688 (8)	0.0034 (5)	0.0347 (6)	0.0067 (5)
O2	0.0614 (7)	0.0684 (8)	0.0369 (6)	0.0013 (6)	0.0112 (5)	0.0091 (5)

### Geometric parameters (Å, °)

**Table:**

C1—N1	1.4771 (17)	C10—H10	0.9300
C1—C2	1.5235 (19)	C11—H11	0.9300
C1—C13	1.5243 (19)	C12—H12A	0.9600
C1—H1	0.9800	C12—H12B	0.9600
C2—C3	1.501 (2)	C12—H12C	0.9600
C2—H2A	0.9700	C13—C18	1.386 (2)
C2—H2B	0.9700	C13—C14	1.389 (2)
C3—O1	1.2123 (16)	C14—C15	1.379 (2)
C3—C4	1.510 (2)	C14—H14	0.9300
C4—C12	1.512 (2)	C15—C16	1.372 (3)
C4—C5	1.5476 (19)	C15—H15	0.9300
C4—H4	0.9800	C16—C17	1.383 (3)
C5—N1	1.4856 (17)	C16—H16	0.9300
C5—C6	1.5179 (19)	C17—C18	1.387 (2)
C5—H5	0.9800	C17—H17	0.9300
C6—C11	1.379 (2)	C18—H18	0.9300
C6—C7	1.384 (2)	C19—O2	1.2172 (18)
C7—C8	1.386 (2)	C19—N1	1.3645 (18)
C7—H7	0.9300	C19—C20	1.532 (2)
C8—C9	1.369 (3)	C20—N2	1.461 (2)
C8—H8	0.9300	C20—H20A	0.9700
C9—C10	1.369 (3)	C20—H20B	0.9700
C9—H9	0.9300	N2—N3	1.233 (2)
C10—C11	1.380 (3)	N3—N4	1.119 (2)
			
N1—C1—C2	107.80 (11)	C10—C11—C6	121.20 (17)
N1—C1—C13	113.16 (11)	C10—C11—H11	119.4
C2—C1—C13	116.63 (12)	C6—C11—H11	119.4
N1—C1—H1	106.2	C4—C12—H12A	109.5
C2—C1—H1	106.2	C4—C12—H12B	109.5
C13—C1—H1	106.2	H12A—C12—H12B	109.5
C3—C2—C1	112.38 (12)	C4—C12—H12C	109.5
C3—C2—H2A	109.1	H12A—C12—H12C	109.5
C1—C2—H2A	109.1	H12B—C12—H12C	109.5
C3—C2—H2B	109.1	C18—C13—C14	118.28 (14)
C1—C2—H2B	109.1	C18—C13—C1	119.39 (13)
H2A—C2—H2B	107.9	C14—C13—C1	122.12 (13)
O1—C3—C2	121.36 (13)	C15—C14—C13	120.68 (17)
O1—C3—C4	122.66 (13)	C15—C14—H14	119.7
C2—C3—C4	115.97 (11)	C13—C14—H14	119.7
C3—C4—C12	112.72 (12)	C16—C15—C14	120.73 (18)
C3—C4—C5	111.22 (12)	C16—C15—H15	119.6
C12—C4—C5	110.61 (12)	C14—C15—H15	119.6
C3—C4—H4	107.3	C15—C16—C17	119.44 (17)
C12—C4—H4	107.3	C15—C16—H16	120.3
C5—C4—H4	107.3	C17—C16—H16	120.3
N1—C5—C6	113.86 (11)	C16—C17—C18	119.93 (18)
N1—C5—C4	111.92 (10)	C16—C17—H17	120.0
C6—C5—C4	110.39 (11)	C18—C17—H17	120.0
N1—C5—H5	106.7	C17—C18—C13	120.90 (17)
C6—C5—H5	106.7	C17—C18—H18	119.6
C4—C5—H5	106.7	C13—C18—H18	119.6
C11—C6—C7	118.25 (14)	O2—C19—N1	121.75 (14)
C11—C6—C5	119.41 (13)	O2—C19—C20	120.57 (13)
C7—C6—C5	122.12 (13)	N1—C19—C20	117.68 (13)
C6—C7—C8	120.36 (16)	N2—C20—C19	111.92 (14)
C6—C7—H7	119.8	N2—C20—H20A	109.2
C8—C7—H7	119.8	C19—C20—H20A	109.2
C9—C8—C7	120.50 (18)	N2—C20—H20B	109.2
C9—C8—H8	119.8	C19—C20—H20B	109.2
C7—C8—H8	119.8	H20A—C20—H20B	107.9
C8—C9—C10	119.64 (16)	C19—N1—C1	122.20 (12)
C8—C9—H9	120.2	C19—N1—C5	115.31 (11)
C10—C9—H9	120.2	C1—N1—C5	117.91 (10)
C9—C10—C11	120.04 (17)	N3—N2—C20	116.64 (14)
C9—C10—H10	120.0	N4—N3—N2	171.04 (17)
C11—C10—H10	120.0		
			
N1—C1—C2—C3	57.25 (15)	N1—C1—C13—C14	−135.96 (14)
C13—C1—C2—C3	−71.29 (15)	C2—C1—C13—C14	−10.06 (19)
C1—C2—C3—O1	167.38 (13)	C18—C13—C14—C15	1.3 (2)
C1—C2—C3—C4	−12.02 (17)	C1—C13—C14—C15	−173.35 (14)
O1—C3—C4—C12	15.3 (2)	C13—C14—C15—C16	0.3 (3)
C2—C3—C4—C12	−165.33 (13)	C14—C15—C16—C17	−1.7 (3)
O1—C3—C4—C5	140.15 (14)	C15—C16—C17—C18	1.6 (3)
C2—C3—C4—C5	−40.45 (16)	C16—C17—C18—C13	−0.1 (3)
C3—C4—C5—N1	47.54 (15)	C14—C13—C18—C17	−1.4 (2)
C12—C4—C5—N1	173.60 (12)	C1—C13—C18—C17	173.41 (14)
C3—C4—C5—C6	175.48 (11)	O2—C19—C20—N2	4.4 (2)
C12—C4—C5—C6	−58.46 (16)	N1—C19—C20—N2	−175.73 (13)
N1—C5—C6—C11	−118.49 (15)	O2—C19—N1—C1	−163.94 (13)
C4—C5—C6—C11	114.64 (15)	C20—C19—N1—C1	16.16 (19)
N1—C5—C6—C7	67.05 (17)	O2—C19—N1—C5	−8.5 (2)
C4—C5—C6—C7	−59.82 (18)	C20—C19—N1—C5	171.63 (12)
C11—C6—C7—C8	−0.9 (2)	C2—C1—N1—C19	104.11 (14)
C5—C6—C7—C8	173.64 (15)	C13—C1—N1—C19	−125.40 (13)
C6—C7—C8—C9	0.4 (3)	C2—C1—N1—C5	−50.76 (15)
C7—C8—C9—C10	0.5 (3)	C13—C1—N1—C5	79.74 (14)
C8—C9—C10—C11	−0.7 (3)	C6—C5—N1—C19	76.01 (15)
C9—C10—C11—C6	0.2 (3)	C4—C5—N1—C19	−157.92 (12)
C7—C6—C11—C10	0.6 (2)	C6—C5—N1—C1	−127.41 (12)
C5—C6—C11—C10	−174.06 (16)	C4—C5—N1—C1	−1.34 (16)
N1—C1—C13—C18	49.44 (17)	C19—C20—N2—N3	−79.48 (19)
C2—C1—C13—C18	175.34 (13)		

### Hydrogen-bond geometry (Å, °)

**Table:**

*D*—H···*A*	*D*—H	H···*A*	*D*···*A*	*D*—H···*A*
C9—H9···O1^i^	0.93	2.57	3.464 (2)	162
C5—H5···N2^ii^	0.98	2.52	3.353 (2)	142
C2—H2B···O2^ii^	0.97	2.56	3.4933 (19)	161

Symmetry codes: (i) −*x*+2, *y*+1/2, −*z*+1/2; (ii) −*x*+1, −*y*, −*z*.
